# Improving the quality of child and youth mental health care through an implementation science lens

**DOI:** 10.3389/frhs.2026.1731708

**Published:** 2026-02-13

**Authors:** Lars Dumke, Jennifer Hall, Jenny Jung, Raffaella Sibilio, Karina Beinerte, Rimma Beļikova, Venetsanos Mavreas, Stelios Stylianidis, Natasha Azzopardi-Muscat, Ledia Lazeri, Brian Oldenburg, João Breda

**Affiliations:** 1 WHO Athens Office on Quality of Care and Patient Safety, WHO Regional Office for Europe, Athens, Greece; 2 Department of Psychiatry and Psychotherapy, University Medical Center Hamburg-Eppendorf, Hamburg, Germany; 3 Department of Public Health and Implementation Science, La Trobe University, and Baker Heart and Diabetes Institute, Melbourne, VA, Australia; 4 Child and Youth Mental Health Centre, Children’s Clinical University Hospital, Riga, Latvia; 5 Methodological Centre, Children’s Clinical University Hospital, Riga, Latvia; 6 Faculty of Medicine, School of Health Sciences, University of Ioannina & EPAPSY, Ioannina, Greece; 7 Department of Psychology, Panteion University & EPAPSY, Athens, Greece; 8 Department of Psychiatry, WHO Regional Office for Europe, Copenhagen, Denmark

**Keywords:** child and adolescent mental health, implementation science, mental health services, quality standards, youth engagement

## Abstract

Mental disorders in children and youth are on the rise worldwide, affecting approximately one in five young people in Europe. The negative educational, social and economic consequences of mental health problems underline the urgent need for accessible, high-quality mental health services. In response, the WHO Regional Office for Europe has developed Quality Standards for Child and Youth Mental Health Services to provide a framework for evidence-based, youth-centred care. Previous efforts have shown that translating quality standards and guidelines into practice remains a major challenge. While implementation science offers valuable approaches to improving the uptake of evidence-based quality standards and has been successfully applied in various healthcare fields, it remains underutilised in child and youth mental health care. This article explores how implementation science can be leveraged to improve the quality of child and youth mental health care. Using the implementation of the WHO European Quality Standards for Child and Youth Mental Health Services as a practical case example, we highlight the importance of a structured approach to implementation, guidance by theories, models and frameworks, timely stakeholder engagement, and context-specific adaptation. Harnessing implementation science in youth mental health policy and practice has the potential to bridge the gap between policy formulation and real-world service delivery, ensuring that all young people receive the high-quality mental health care they deserve.

## Introduction

The mental health of children and youth in the World Health Organization (WHO) European Region is deteriorating (1). In this region, approximately one in five young people aged 15–19 years lives with a mental disorder (2, 3). Mental health support is most effective when it is received timely and is of high quality. Limited access to quality mental health care can have lifelong negative impacts on young people, their families, and communities. Across the WHO European Region, mental health conditions are the leading cause of disability among young people, emphasising the need to improve access and quality of care (4).

However, despite increasing recognition of child and youth mental health as a public health priority, significant disparities exist in access, quality, and outcomes of mental health services across the WHO European Region. More than one-third of countries in the WHO European Region lack mental health policies or plans for children and adolescents (5), and just over half have mechanisms in place to assess the quality of child and adolescent mental health services (6). Standardizing and improving service quality is crucial to reducing existing inequalities and ensuring all children and youth receive the mental health care they need.

## WHO Europe’s approach to improving quality in mental health care for children and youth

To address these gaps, the WHO Regional Office for Europe developed Quality Standards for Child and Youth Mental Health Services (the *‘Standards’*), providing a comprehensive framework for delivering evidence-based, accessible, and youth-centred care (7). The development of the *Standards* followed a collaborative, evidence-guided process which actively engaged a wide range of collaborators, including children and youth, their carers, and service providers. The development process was informed through a comprehensive review of national and international quality standards and peer-reviewed literature, as well as input and contributions from service users and providers. This included surveys, focus group discussions, and consultations to identify what constitutes good quality mental health care from the perspective of young people and those who support them (7).

The resulting *Standards* outline essential components for high-quality mental health care: (1) Youth participation and empowerment, (2) Rights and safety, (3) Family and community engagement, (4) Smooth transitions, (5) Timely support, (6) Developmentally appropriate and evidence-based support, (7) Workforce competency, and (8) Quality improvement and data collection. The *Standards* are intended to serve as a benchmark for governments, policymakers, and service providers to assess and improve mental health systems. Their overarching goal is to ensure that all children and young people, regardless of their background or location, receive high-quality mental health care that is safe, effective, and equitable.

## Implementation science: a crucial but under-recognised field in mental health care

While the development of quality standards is a critical step, their existence does not guarantee better service delivery or improved outcomes. Research shows that guidelines and standards are not always effectively implemented and often they remain underutilised or inconsistently applied (8). It is estimated that only 60% of healthcare is provided according to evidence-based guidelines, while 30% is unnecessary or of low value, and 10% causes harm (9). In child and youth mental health care, adherence to guidelines is similarly low (10). This may be due to several barriers, including a lack of appropriate implementation strategies and stakeholder involvement, which compromises the incorporation of context-specific knowledge and expertise (10). Poor guideline implementation may help explain why WHO World Mental Health Survey data show that at least two-thirds of patients with major depressive or anxiety disorders in high-income countries receive inadequate treatment (11, 12).

The persistent gap between what is known, the provision of adequate care and what is done in practice (i.e., the ‘knowledge-implementation gap’) emphasises the need for systematic approaches to support the implementation of evidence-based practice. Bridging this gap requires more than producing guidelines and standards; it demands a focused effort to understand the implementation process and to develop strategies to sustainably embed quality standards into everyday care (13).

Implementation science provides systematic methods and strategies to facilitate the integration of evidence-based practices into routine care. It focuses on translating research into practice, identifying barriers to implementation, and ensuring sustainable change through evaluation. A wide range of models, frameworks, and theories provide guidance in optimizing implementation success (14).

Implementation science has been previously applied to improve quality of care, enhance patient safety, implement clinical guidelines, and facilitate many different health innovations (15–18). However, its role in enhancing quality improvement efforts in mental health care, particularly in child and youth mental health care, remains largely under-investigated (19–21), with only a few examples of evaluations of quality improvement in child and adolescent mental health service found (22, 23). The implementation of quality standards in particular in this field has received little attention, as highlighted by a 2023 systematic review on the implementation of health and social care standards, which found no studies addressing quality standards for youth mental health (20).

Without a thorough examination and planning of the implementation process, adopting and sustaining quality standards is unlikely. Successful implementation of quality standards requires a comprehensive understanding of key factors, including who is responsible for implementation, the cultural and systemic contexts in which it takes places, and perceptions and attitudes toward implementation (20). Moreover, for successful implementation efforts, engaging a broad range of partners in sustained, long-term collaborations is essential (24). This involves guideline and standards developers working closely with implementers, such as policymakers, service providers and users, as well as implementation researchers, to ensure that the implementation process is contextually relevant and addresses the actual needs and challenges faced in practice (8). Systematic consideration of these factors is crucial for developing targeted strategies to strengthen implementation and ultimately improve the quality and safety of mental health care. While implementation science provides useful tools, there is limited experience of its application in the context of child and youth mental health policy. Therefore, capturing and systematically documenting how implementation science approaches are used to facilitate the uptake of new guidelines and standards is critical to informing future implementation efforts and identifying effective strategies to improve their uptake.

Here we describe how implementation science has been utilised to enhance the implementation and uptake of the WHO Quality Standards for Child and Youth Mental Health Services.

## Harnessing implementation science: a case example of the WHO regional office for Europe quality standards for child and youth mental health services

### Timely development of implementation support and resources

From the outset, the *‘Standards’* development and implementation process aimed to capitalise on early motivation and opportunity. This involved the use of the Capability, Opportunity, and Motivation Model of Behaviour (COM-B Model), a behaviour change framework that provides insights into an organisation's readiness for implementation (25). The COM-B Model proposes that readiness to implement a change depends on capability, opportunity and motivation. For the *Standards,* it was therefore essential to target these factors among decision makers and implementers. When a product is launched, the initial excitement and interest can create a window of opportunity and motivation for its implementation. In case implementation support is not available, this excitement can quickly dissipate, and motivation and opportunity lost. We aimed to overcome this barrier by simultaneously developing and launching the *Standards* alongside tools to support its implementation. A rapid review of implementation tools for standards and guidelines was conducted as soon as the idea for the *Standards* had been conceptualised. Initial versions of the implementation tools were developed at the same time as the *Standards*, allowing for a parallel consultation and update process. Upon development of initial versions of the *Standards* and its implementation tools were available, interviews with key stakeholders were conducted to understand key implementation barriers and facilitators and provide further support where necessary. This enabled lessons already learnt on implementation processes to be available for those interested in implementing the *Standards* at the time of their launch.

To strengthen the perceived capability of policymakers and service providers, case studies illustrating the practical implementation of the *Standards* were developed. These case studies demonstrated how different stakeholders could adapt and integrate the *Standards* into their existing practice. By highlighting real-world applications, they provided tangible examples that implementation was achievable. The case studies were shared widely among networks and will be made available online.

### Interdisciplinary collaboration

A key aspect in harnessing the potential of implementation science was the collaboration between a diverse array of experts in implementation science, child and youth mental health, and quality of care. Through regular meetings, joint workshops, and iterative discussions, the team created spaces to exchange perspectives and challenge assumptions about implementation approaches. Implementation scientists helped to ensure that approaches are grounded in evidence-based frameworks, theories and models, while mental health professionals contributed contextual knowledge of service delivery and settings across the WHO European Region, and quality of care experts contributed experience of having implemented quality improvement in health systems. This interdisciplinary approach facilitated the integration of evidence-informed implementation methods while ensuring relevance to children and youth mental health care settings.

### Engaging service users, providers, and decision-makers

Given the significant heterogeneity among the 53 Member States in the WHO European Region, ensuring relevant implementation support across various health system contexts was a major challenge. Efforts to overcome this barrier included actively involving a wide diversity of partners and member states from across the WHO European Region. The implementation process of the *Standards* engaged international organizations, policymakers, program managers, health care professionals, as well as service users and their carers throughout all stages. The implementation of the *Standards* was discussed and refined through feedback gathered at key events and conferences, including the 3rd WHO Mental Health Week (2–7 November 2024), with over 200 participants, and the 1st WHO Autumn School on Quality of Child and Adolescent Mental Health (2–6 November 2024), which brought together 36 participants from 15 different countries. There were continuous efforts to build multi-sectoral and cross-country partnerships. We used embedded research approaches to integrate decision makers and implementers and establish a direct link to the use of findings. For instance, the developed implementation tools were shared widely across the whole WHO European Region for feedback decision makers and implementers and updated accordingly. Moreover, in-depth interviews were held across 4 countries (Latvia, Greece, North Macedonia, and Kazakhstan), purposefully selected as ‘early adopters’ due to their heterogeneous nature, to inform context-specific adaptations and increase the potential for successful implementation across the Region.

### Consideration of implementation needs across a multi-sectoral system

Implementation theories identify the importance of engaging different levels of the health system for successful implementation. Applied to a mental health system, this can mean engaging those responsible for governance and leadership, as well as those responsible for quality audits and improvement at the service level. In recognition of its holistic nature, a multi-sectoral approach is recommended for child and youth mental health care with smooth transitions and working between different sectors (7, 26). To gain a comprehensive perspective on the implementation of the *Standards*, a diverse group of collaborators, including policymakers, service managers, and service providers from each country were interviewed to assess knowledge, awareness, perception, and implementation factors (such as adoption, capacity, adaptation, facilitators, and barriers) of the *Standards* across all layers of the multi-sectoral system. This was guided by the Consolidated Framework for Implementation Research (CFIR), which offers a comprehensive structure to assess factors influencing implementation outcomes (27).

Interviews across countries revealed common barriers to implementation, including workforce capacity constraints and resource limitations. Facilitators included strong multi-sectoral collaboration, sustainability through long-term policy commitment and financial investment, and involvement of the perspectives of young people and their families. Most collaborators agreed that adopting the *Standards* would be difficult without context-specific adaptations such as translation of resources, considering sociocultural factors, and aligning outputs with available resources. Key findings from the interviews were shared at a technical meeting for further discussion. Participants felt that their perspective had been well taken into account and found the findings useful as a basis for deriving context-specific implementation actions, designing implementation plans, and planning capacity-building activities.

## Moving forward to improve child and youth mental health

The learnings from the implementation process of the *Standards* illustrate the potential of implementation science in advancing child and youth mental health care. In line with evidence from other health sectors, applying structured and systematic methods can help anticipate and overcome potential barriers, thereby ensuring more efficient and sustainable implementation of quality standards, guidelines, and service innovations (15, 16, 18). Individuals and organizations involved in policy, research, and practice can benefit from more systematically applying implementation science to improve child and youth mental health care.

A key factor for successful implementation is the meaningful engagement of all individuals and groups affected by the process or its intended outcomes. When policymakers, researchers, service providers, and service users are actively involved, mental health services become more relevant, effective, and of higher quality (7). Our experience highlights the importance of engaging service users, community members, implementers, and decision-makers early on and sustaining their involvement throughout the implementation process. In the context of child and youth mental health care, this is particularly important as young people are often excluded from decision-making processes that directly affect them (28).

Moreover, to effectively implement quality standards, broader societal barriers must be addressed, as decision makers and implementers also highlighted in the implementation of the *Standards*. This may include tackling mental health stigma, raising public awareness through innovative and youth-engaging communication strategies, and shifting from top-down approaches to community-driven solutions. Social participation is vital for building resilient and sustainable health systems in the European Region (29), making it crucial to provide space, listen to, and act upon the voices of children, youth, and their families. We strongly call for more meaningful and inclusive participation of young people in implementation and quality improvement efforts in mental health care to ensure that services truly reflect their needs and lived realities (30).

Successful implementation efforts require more than technical guidance; long-term quality improvement depends on strong political commitment and sustained investment. Given the worsening state of youth mental health in the WHO European Region (1, 3), it is essential that policymakers champion efforts to address persistent challenges such as geographical disparities in service availability, inadequate funding and/or resource allocation, and a shortage of trained professionals in child and youth mental health. Research, particularly implementation science, plays a key role in providing the necessary evidence to inform and guide these efforts (19). Without targeted action on system-level barriers, quality standards risk remaining aspirational rather than transformational.

Looking ahead, greater emphasis on sharing methods, good practices, challenges, and lessons learned across countries will be essential for advancing child and youth mental health care. This can be facilitated through communities of practice, strategic communication efforts, and other collaborative platforms. Such approaches could generate valuable insights and send strong signals to support policymakers and service leaders in driving quality improvements.

## Conclusion

While the *Standards* provide an innovative evidence-based framework for improving child and youth mental health services, their impact on mental health and wellbeing of young people depends on effective implementation. Through the systematic use of implementation science, WHO aims to bridge the gap between governance and practice to ensure that all young people receive the high-quality mental health care they need and deserve. The lessons learned from this case study echo learnings from previous initiatives, such as the importance of adopting an implementation science lens at all stages, the use of structured methodologies, strong and sustained stakeholder collaboration, and the continuous adaptation of implementation to different contexts. In addition, the learnings highlight the importance of taking a multi-sectoral approach that engages children, young people and their caregivers in the process in order to develop strategies that are grounded in the context of child and youth mental health care to be developed. Drawing on these lessons can help optimise future implementation initiatives in health care for greater sustainability and impact. Fostering collaborative communities of practice and meaningfully engaging young people can advance evidence-based improvements in child and youth mental health care in the WHO European Region and beyond.

## Data Availability

The original contributions presented in the study are included in the article/Supplementary Material, further inquiries can be directed to the corresponding author.

## References

[1] McGorry PD Mei C Dalal N Alvarez-Jimenez M Blakemore S-J Browne V The lancet psychiatry commission on youth mental health. Lancet Psychiatry. (2024) 11(9):731–74. 10.1016/S2215-0366(24)00163-9 39147461

[2] IHME. Global Burden of Disease Study 2021. Seattle, WA: Institute for Health Metrics and Evaluation (IHME), University of Washington (2024).

[3] WHO Regional Office for Europe. Child and Youth Mental Health in the WHO European Region: Status and Actions to Strengthen the Quality of Care. Copenhagen: WHO Regional Office for Europe (2025). Licence: CC BY-NC-SA 3.0 IGO.

[4] Castelpietra G Knudsen AKS Agardh EE Armocida B Beghi M Iburg KM The burden of mental disorders, substance use disorders and self-harm among young people in Europe, 1990–2019: findings from the global burden of disease study 2019. Lancet Regional Health Europe. (2022) 16:100341. 10.1016/j.lanepe.2022.100341 35392452 PMC8980870

[5] WHO. Mental Health ATLAS 2020. Geneva: World Health Organization (2021). Licence: CC BY-NC-SA 3.0 IGO.

[6] WHO. Situation of Child and Adolescent Health in Europe. Copenhagen: World Health Organization, Regional Office for Europe (2018).

[7] WHO Regional Office for Europe. Quality Standards for Child and Youth Mental Health Services: For use in Specialized Community or Outpatient Care Across the WHO European Region. Copenhagen: WHO Regional Office for Europe (2025). Available online at: https://iris.who.int/handle/10665/380778 (Accessed January 30, 2026). Licence: CC BY-NC-SA 3.0 IGO

[8] Saluja K Reddy KS Wang Q Zhu Y Li Y Chu X Improving WHO’s understanding of WHO guideline uptake and use in member states: a scoping review. Health Research Policy Syst. (2022) 20(1):98. 10.1186/s12961-022-00899-y PMC9449928 36071468

[9] Braithwaite J Glasziou P Westbrook J. The three numbers you need to know about healthcare: the 60-30-10 challenge. BMC Med. (2020) 18(1):102. 10.1186/s12916-020-01563-4 32362273 PMC7197142

[10] Westerlund A Ivarsson A Richter-Sundberg L. Evidence-based practice in child and adolescent mental health services—the challenge of implementing national guidelines for treatment of depression and anxiety. Scand J Caring Sci. (2021) 35(2):476–84. 10.1111/scs.12859 32323362

[11] Alonso J Liu Z Evans-Lacko S Sadikova E Sampson N Chatterji S Treatment gap for anxiety disorders is global: results of the world mental health surveys in 21 countries. Depress Anxiety. (2018) 35(3):195–208. 10.1002/da.22711 29356216 PMC6008788

[12] Vigo D Haro JM Hwang I Aguilar-Gaxiola S Alonso J Borges G Toward measuring effective treatment coverage: critical bottlenecks in quality- and user-adjusted coverage for major depressive disorder. Psychol Med. (2022) 52(10):1948–58. 10.1017/S0033291720003797 PMC9341444 33077023

[13] Wang Z Norris SL Bero L. Implementation plans included in world health organisation guidelines. Implement Sci. (2015) 11(1):76. 10.1186/s13012-016-0440-4 PMC4875699 27207104

[14] Nilsen P. Making sense of implementation theories, models and frameworks. Implement Sci. (2015) 10(1):53. 10.1186/s13012-015-0242-0 25895742 PMC4406164

[15] Braithwaite J Marks D Taylor N. Harnessing implementation science to improve care quality and patient safety: a systematic review of targeted literature. Int J Qual Health Care. (2014) 26(3):321–9. 10.1093/intqhc/mzu047 24796491

[16] Chaudoir SR Dugan AG Barr CH. Measuring factors affecting implementation of health innovations: a systematic review of structural, organizational, provider, patient, and innovation level measures. Implement Sci. (2013) 8(1):22. 10.1186/1748-5908-8-22 23414420 PMC3598720

[17] Fisher G Smith CL Pagano L Spanos S Zurynski Y Braithwaite J. Leveraging implementation science to solve the big problems: a scoping review of health system preparations for the effects of pandemics and climate change. Lancet Planetary Health. (2025) 9(4):e326–36. 10.1016/S2542-5196(25)00056-7 40252679

[18] Hakkennes S Dodd K. Guideline implementation in allied health professions: a systematic review of the literature. Qual Safety Health Care. (2008) 17(4):296–300. 10.1136/qshc.2007.023804 18678729

[19] Forman-Hoffman VL Middleton JC McKeeman JL Stambaugh LF Christian RB Gaynes BN Quality improvement, implementation, and dissemination strategies to improve mental health care for children and adolescents: a systematic review. Implement Sci. (2017) 12(1):93. 10.1186/s13012-017-0626-4 28738821 PMC5525230

[20] Kelly Y O’Rourke N Flynn R O’Connor L Hegarty J. Factors that influence the implementation of (inter)nationally endorsed health and social care standards: a systematic review and meta-summary. BMJ Qual Saf. (2023) 32(12):750–62. 10.1136/bmjqs-2022-015287 37290917 PMC10803983

[21] Tarasenko A Josy G Minnis H Hall J Danese A Lau JYF Mental health of children and young people in the WHO Europe region. Lancet Reg Health Europe. (2025) 57:101459. 10.1016/j.lanepe.2025.101459 41132774 PMC12541636

[22] Banwell E Humphrey N Qualter P. Reformed child and adolescent mental health services in a devolved healthcare system: a mixed-methods case study of an implementation site. Front Health Serv. (2023) 3:1112544. 10.3389/frhs.2023.1112544 37213205 PMC10196272

[23] Stafford J Aurelio M Shah A. Improving access and flow within child and adolescent mental health services: a collaborative learning system approach. BMJ Open Qual. (2020) 9(4):e000832. 10.1136/bmjoq-2019-000832 33208305 PMC7677356

[24] Biermann O Schleiff M Romao DMM Mikaelsdotter C Alfvén T Wanyenze RK Action towards connecting knowledge translation and implementation research. Lancet Glob Health. (2025) 13(3):e403–4. 10.1016/S2214-109X(24)00522-9 39929226

[25] Michie S Van Stralen MM West R. The behaviour change wheel: a new method for characterising and designing behaviour change interventions. Implement Sci. (2011) 6(1):42. 10.1186/1748-5908-6-42 21513547 PMC3096582

[26] WHO and UNICEF. Mental Health of Children and Young People: Service Guidance. Geneva: World Health Organization (2024). Licence: CC BY-NC-SA 3.0 IGO.

[27] Damschroder LJ Reardon CM Widerquist MAO Lowery J. The updated consolidated framework for implementation research based on user feedback. Implement Sci. (2022) 17(1):75. 10.1186/s13012-022-01245-0 36309746 PMC9617234

[28] Yamaguchi S Bentayeb N Holtom A Molnar P Constantinescu T Tisdall EKM Participation of children and youth in mental health policymaking: a scoping review [part I]. Admin Policy Mental Health Mental Health Serv Res. (2023) 50(1):58–83. 10.1007/s10488-022-01223-0 36357819

[29] Azzopardi Muscat N Kluge HHP. A framework for resilient and sustainable health systems in the European region. Lancet Reg Health Europe. (2025) 49:101199. 10.1016/j.lanepe.2024.101199 39831146 PMC11741075

[30] Hall J Nesrallah S Ras D Bailie A Brunskill H Jutten E Youth participation in strengthening the quality of child, adolescent, and youth mental health in the World Health Organization European region. J Adolesc Health. (2024) 75(3):519–21. 10.1016/j.jadohealth.2024.04.025 39152011

